# Detection of hepatocellular carcinoma methylation markers in salivary DNA

**DOI:** 10.1042/BSR20232063

**Published:** 2024-03-21

**Authors:** Catherine Mezzacappa, Zhanwei Wang, Lingeng Lu, Harvey Risch, Tamar Taddei, Herbert Yu

**Affiliations:** 1 Department of Internal Medicine, Yale School of Medicine, New Haven, CT, United States; 2 University of Hawai’i Cancer Consortium, Honolulu, HI, United States; 3 Department of Chronic Disease Epidemiology, Yale School of Public Health, New Haven, CT, United States; 4 VA Connecticut Healthcare System, West Haven, CT, United States

**Keywords:** biomarkers, hepatocellular carcinoma, methylation, screening

## Abstract

Background: Alterations to DNA methylation have been identified in both hepatocellular carcinoma (HCC) tumor and circulating DNA from affected individuals. These markers have potential utility in HCC screening. Adherence to HCC screening is poor and acceptable HCC screening tests are needed.

Methods: A feasibility study was performed on a subset of case patients and control subjects from a prior study of risk factors for HCC. Case patients (*n*=12) included adults aged 47–85 years with a first diagnosis of HCC between 2011 and 2016 and without viral hepatitis. Control subjects (*n*=12) were matched on age, sex, and state of residence. Participants provided saliva samples for DNA genotyping. Log fold change in salivary DNA methylation at 1359 CpG sites representing 25 candidate genes previously associated with HCC was compared across case patients and control subjects.

Results: The quantity of DNA ranged from 9.65 to 257.79 μg. The purity of DNA isolates was good, with mean OD260/280 ratio of 1.78 (SD: 0.14). Of 25 candidate genes, 16 had at ≥1 CpG site with detectable differences in methylation across HCC case patients and control subjects. Sites differentially methylated in HCC case patients included genes encoding tumor suppressors (PRDM2, RUNX3, p15/16, and RASSF1/5), regulators of cell cycle progression (DAPK1 and TP73), and DNA repair (MGMT and GSTP1). No associations met the significance threshold 3.7 × 10^−5^ required for multiple comparisons.

Conclusions: Salivary DNA may be a feasible alternative to blood samples in the era of novel DNA-based screening tests for HCC. The ease of saliva-based testing supports further investigation of its potential.

## Background

Hepatocellular carcinoma (HCC) is a leading cause of mortality in patients with cirrhosis. The epidemiology of HCC is changing with the availability of effective treatments for hepatitis C virus and the rising prevalence of metabolic disease and its phenotype in the liver, metabolic dysfunction-associated steatotic liver disease (MASLD). While HCC rates in MASLD are lower than those observed in other liver diseases including alcohol and HCV-associated cirrhosis, the fraction of HCC cases attributable to MASLD is on the rise [1,2].

Currently, the American Association for the Study of Liver Diseases (AASLD) recommends offering screening for HCC with an ultrasound or contrast-enhanced CT or MRI and a serum α-fetoprotein (AFP) every 6 months when HCC risk is at least 1.5% per year, which includes persons with established cirrhosis and chronic hepatitis B virus infection [3]. Guidelines do not recommend screening in patients with MASLD without cirrhosis, and HCC risk stratification in this large population remains a clinical and population health dilemma [4]. The sensitivity and specificity of ultrasound and AFP for HCC screening are sub-optimal and efforts are underway to develop novel biomarkers for use in HCC screening, including combination DNA methylation assays [5,6].

Numerous epigenetic alterations have been identified in HCC tissue [7,8]. Prior research has analyzed HCC tumor [8–14] or compared blood samples from persons with HCC to persons with chronic viral hepatitis [15,16]. Circulating tumor DNA (ctDNA) in peripheral blood samples is undergoing evaluation as a screening test for HCC [5,6]. A recent study evaluated urinary ctDNA and found a combination of ctDNA markers from urine added to the accuracy of HCC screening among persons with low serum AFP [17]. Saliva is more comfortable and easier for patients to collect than other body fluids and may serve as a more acceptable test to perform on a repeated basis for screening purposes [18–20].

Saliva has been used to identify DNA from head and neck cancers [21] and lung cancers [22]. Tumors in sites not in communication with the oropharynx have not been as deeply examined, but the close interactions between capillary beds and salivary glands suggest examining ctDNA in saliva is achievable with adequate measures to ensure sample quality [23–25]. The aim of this study was to evaluate the feasibility of detecting aberrant DNA methylation patterns previously identified in HCC tumor and peripheral blood in saliva.

## Methods

This is an observational feasibility study performed on a convenience sample of individuals with adequate stored DNA samples identified from a previously conducted case–control study of genetic and environmental risk factors for HCC [26]. Case patients included in the present study were individuals aged 47–85 years with a first diagnosis of HCC and without a history of viral hepatitis diagnosed between January 2011 and February 2016 in Connecticut, New Jersey, or New York City. Control subjects were identified using random digit dialing and included adults without any form of cancer and matched to case patients on age, sex, and state of residence. Additional details regarding participant recruitment are available in Shen et al., 2020 [26]. All study participants completed structured questionnaires by telephone interview and were instructed to mail in saliva samples collected with commercial kits for both DNA genotyping and HCV antibody assay. The saliva sample for genotyping was collected with the Saliva Self-Collection Kit (OG-250, DNA Genotek, Ottawa, Ontario, Canada). Individuals with HCC were recruited to the original case–control study at the time of first HCC diagnosis, and a saliva sample was collected from each enrolled HCC case shortly after diagnosis and prior to HCC treatment.

Information on history of hepatitis C virus (HCV) was ascertained through both questionnaire inquiry and saliva testing. Among individuals with adequate stored DNA for methylation microarray analysis, 12 case patients and 12 control subjects matched on sex, race, and ethnicity without a history of viral hepatitis were selected from the original study for analysis. Participants were selected such that half (6 case patients and 6 control subjects) reported a diagnosis of metabolic dysfunction associated steatotic liver disease (MASLD).

Candidate genes were identified from existing literature on differences in DNA methylation observed in HCC tumor and blood. Ultimately, 25 candidate genes were selected for comparison and are demonstrated in Table 1. The log-fold change in DNA methylation at 1359 CpG sites representing these 25 candidate genes was compared across HCC status in the total sample and in the MASLD subset.

**Table 1:** Candidate genes identified for comparison across HCC case patients and control subjects

Gene	Function
APC	Tumor suppressor
CDH1	E-cadherin
CDKN2A	Tumor suppressor
CDKN2B	Tumor suppressor
DAPK1	Programmed cell death
DRD4	Dopamine receptor
EFNB2	Epithelial–mesenchymal signaling
FAM196A	Rho GTPase
FOXE3	Transcription factor
GSTP1	Metabolic injury
IGF2	Growth factor
MGMT	DNA repair
MLH1	DNA repair
NKX6-2	Transcription factor, cellular differentiation
PRDM2	Tumor suppressor
RARB	Nuclear thyroid hormone receptor
RASSF1	Tumor suppressor
RASSF5	Tumor suppressor
RUNX3	Transcription factor
SFRP1	Wnt signaling modulator
SFRP5	Wnt signaling modulator
SOCS1	STAT-induced STAT inhibitor
TBX15	Transcription factor
TP73	p53 family transcription factor
WIF1	Wnt inhibitor, cell cycle progression

Saliva samples collected for genotyping were processed according to manufacturer instructions [27]. The concentration of DNA in the supernatant, total mass of DNA, and purity of the nucleic acids were assessed for each sample. Nucleic acid purity was measured using the ratio of absorbance at 260 and 280 nm using Thermo Fisher Scientific NanoDrop 1000 spectrophotometry [28]. A ratio of approximately 1.8 is accepted as ‘pure’ for DNA [28,29].

**Figure 1:**
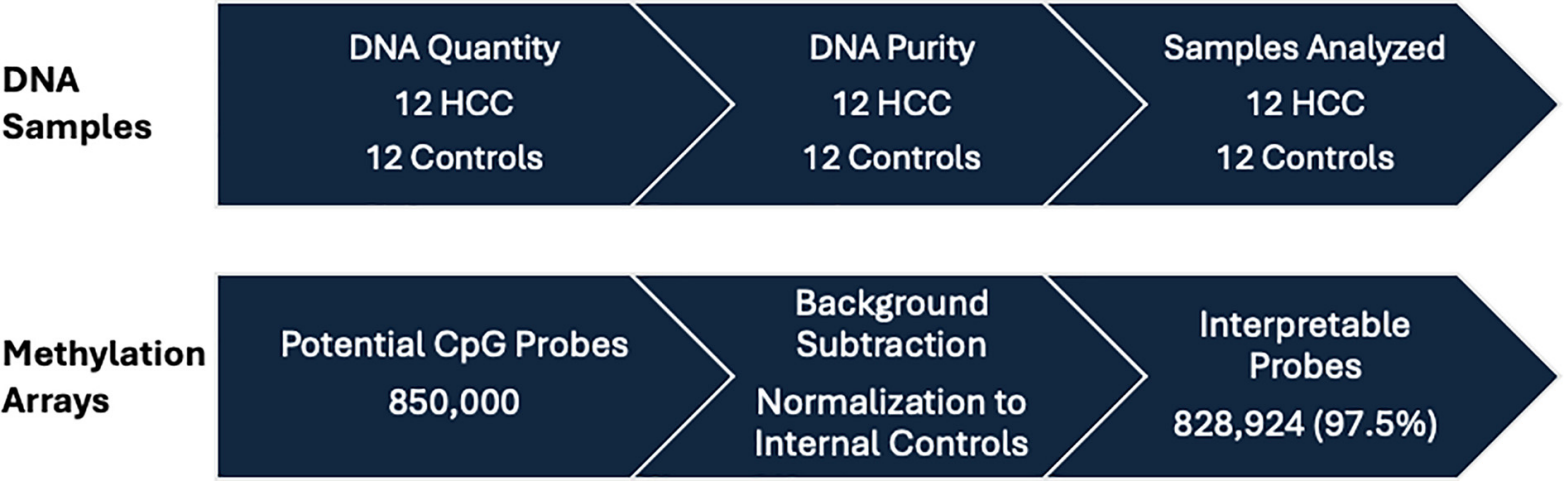
Visual summary of sample identification and DNA quality control

After sodium bisulfite conversion (which converts unmethylated cytosine residues to uracil residues), the methylation profile of salivary DNA was assessed using Illumina Infinium MethylationEPIC (850k) BeadChip methylation arrays. These arrays cover more than 850,000 CpG sites genome-wide. Samples were processed according to the manufacturer’s instructions [30]. Briefly, The Zymo Research EZ DNA methylation kit was used for bisulfate conversion of genomic DNA. Approximately 1000 ng (1 μg) of DNA was diluted in 22 μl of elution buffer and the DNA was denatured and underwent bisulfite conversion per manufacturer instructions. Then, 20 μl of the bisulfate-converted DNA solution was used for whole genome amplification, fragmentation, precipitation, and resuspension prior to hybridization onto the BeadChips. The Illumina iScan SQ System was used to scan BeadChips and create image files, which were extracted using R package minfi. The detectionP function was used to filter any samples and probes not meeting quality control metrics. All samples passed standard quality control metrics. Any probe that failed in ≥1 sample was labeled as not interpretable and removed from the analytic dataset. The data were normalized using background subtraction and normalization to internal controls methods. Internal control beads in each channel were used to set the background probe intensity level at the 5th percentile of the negative controls in the given channel. This background intensity was then subtracted from probe intensities in the same channel (to a minimum of 0). The probe intensity in each sample was then normalized to the probe intensity derived from these internal control probes for all samples. Potential batch effects are addressed through this normalization to internal controls. This process is summarized in Figure 1. Resultant β values for each CpG range from 0 (fully unmethylated) to 1 (fully methylated). These are transformed to the logit of the β values, known as the *M* value, and the log_2_-fold change in *M* values comparing HCC case patients to control subjects was calculated and compared using Limma’s differential analysis, which uses moderated *t*-tests. This moderated *t*-test utilizes information from all CpG sites to inform the variance estimation for each individual site, which improves the reliability of the variance estimates for statistical inference. To account for multiple comparisons, the significance threshold was set at 3.7 × 10^−5^ (0.05/1359).

## Results

The mean OD260/280 ratio of the DNA samples was 1.78 (SD: 0.14). The quantity of DNA varied across samples and ranged from 9.65 to 257.785 μg (Table 3). All samples had adequate quantity of DNA for methylation microarray assays. Of 850,000 total CpG probes, 828,924 (97.5%) had interpretable probe intensities (Figure 1).

**Table 2:** Sample characteristics

Characteristic^*^	Total sample	HCC	No HCC
**Age (median)**	63 (60–67)	67 (65–69)	59 (55–62)
**Male sex**	24 (100%)	12 (100%)	12 (100%)
**Non-Hispanic White race**	24 (100%)	12 (100%)	12 (100%)
**College or more**	17 (70.8%)	7 (58.3%)	10 (83.3%)
**BMI (median)**	29.8 (25.5–33.1)	30.8 (28.5–33.1)	26.7 (24.5–33.5)
**Diagnosed with MASLD**	12 (50.0%)	6 (50.0%)	6 (50.0%)
**Any cigarette smoking**	15 (62.5%)	10 (83.3%)	5 (41.7%)
**Any alcohol use**	17 (70.8%)	11 (91.7%)	6 (50.0%)

* Presented as *n* (%) except for age and BMI, which are presented as median (interquartile range).

**Table 3:** Concentration, quantity, and quality of DNA isolates

Sample	Concentration DNA (ng DNA/μl)	Total DNA (μg)	Nucleic acid purity OD260/280
1	45.77	22.89	1.69
2	233.77	116.90	1.89
3	23.36	11.68	1.65
4	278.84	139.42	1.85
5	287.95	143.98	1.8
6	196.54	98.27	1.75
7	229.35	114.68	1.69
8	337.58	168.79	1.97
9	515.57	257.79	1.76
10	165.94	82.97	1.75
11	356.03	178.02	1.8
12	108.99	54.50	1.32
13	291.79	145.90	1.87
14	19.3	9.65	1.73
15	117.34	58.67	1.59
16	323.46	161.73	1.82
17	265.78	132.89	1.79
18	303.46	151.73	1.73
19	428.64	214.32	1.83
20	313.28	156.64	1.86
21	328.56	164.3	1.94
22	88.32	44.2	1.94
23	143.21	71.605	1.95
24	125.25	62.625	1.81
**Mean**	**230.34**	**115.17**	**1.78**
**SD**	**128.92**	**64.46**	**0.14**

Of the 25 candidate genes identified, 16 had at least one CpG site with a detectable difference in DNA methylation. At the gene level, we would expect between 1 and 2 genes to differ by chance alone assuming an alpha of 0.05 (0.05 × 25).

**Figure 2:**
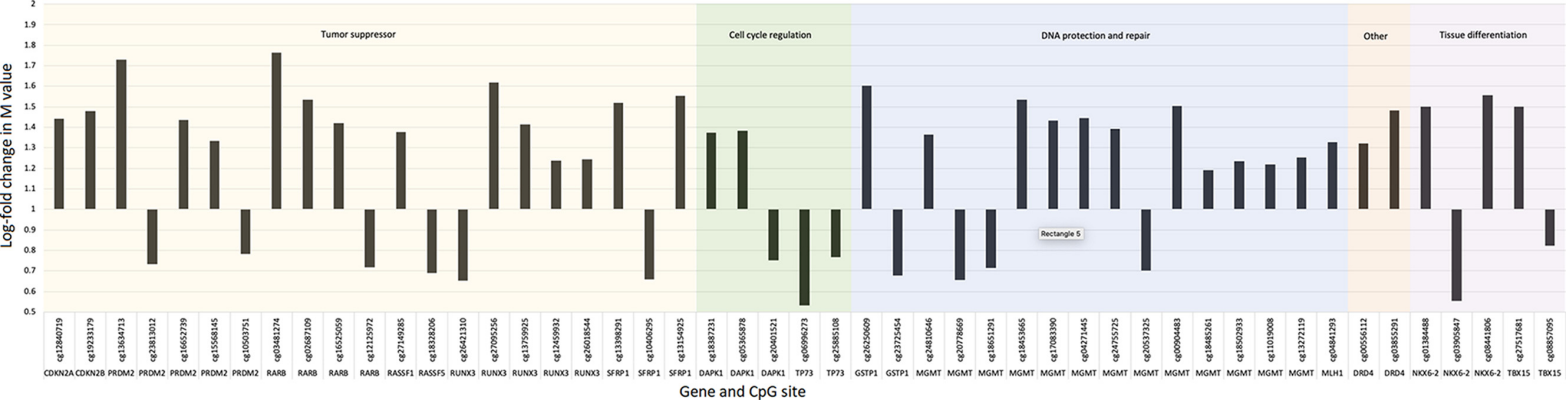
Log-fold difference in DNA methylation by gene function, gene, and CpG site The *y*-axis is set at a value of 1. Bars above 1 are CpG sites that, on average, demonstrated an increase in methylation in cases of hepatocellular carcinoma compared with controls. Bars below 1 are CpG sites that, on average, demonstrated a decrease in methylation in cases of hepatocellular carcinoma compared with controls. The CpG sites are organized by gene function, with gene name along the *x*-axis below the CpG site label.

Individual CpG sites differentially methylated in HCC case patients included genes encoding tumor suppressors (APC, RUNX3, RAR-β, PRDM2, SFRP1, RASSF1A, and RASSF5), regulators of cell cycle progression and death (DAPK1 and TP73), and DNA repair (MGMT, GSTP1, and MLH1) (Tables 4 and 5, Figure 2). The strongest suggestive associations were observed for CpG sites located in NKX6-2, a gene involved in tissue differentiation (log-fold difference in methylation: 1.50, *P*=8.8 × 10^−4^), SFRP1 (log-fold difference in methylation: 1.52, *P*=3.5 × 10^−3^), and MGMT (log-fold difference in methylation: 1.36, *P*=6.3 × 10^−3^). Hypermethylation, demonstrated by these log-fold difference values >1 comparing HCC case patients with control subjects, down-regulates gene transcription. None of the associations measured met the significance threshold of 3.7 × 10^−5^ required to adjust for 1359 comparisons.

**Table 4:** Differences in DNA methylation by gene and CpG site

Gene	CpG	Cases		Controls		Fold difference
		Beta	*M*	Beta	*M*	
PRDM2	cg23813012	0.0289	-5.099	0.0358	-4.788	0.732
PRDM2	cg10503751	0.0842	-3.461	0.0982	-3.218	0.784
PRDM2	cg15568145	0.8415	2.426	0.8127	2.139	1.332
PRDM2	cg16652739	0.7983	2.028	0.7583	1.666	1.436
PRDM2	cg13634713	0.0594	-3.995	0.0452	-4.542	1.729
RUNX3	cg26421310	0.0589	-4.019	0.0794	-3.594	0.654
RUNX3	cg12459932	0.9370	3.907	0.9276	3.694	1.237
RUNX3	cg26018544	0.7912	1.933	0.7656	1.715	1.244
RUNX3	cg13759925	0.8317	2.332	0.7965	1.986	1.414
RUNX3	cg27095256	0.0428	-4.522	0.0320	-5.003	1.618
RARB	cg12125972	0.7966	1.991	0.8303	2.322	0.718
RARB	cg16525059	0.7762	1.801	0.7272	1.450	1.421
RARB	cg02687109	0.7309	1.482	0.6734	1.054	1.535
RARB	cg03481274	0.0889	-3.412	0.0633	-3.980	1.765
SFRP1	cg10406295	0.0415	-4.556	0.0557	-4.141	0.660
SFRP1	cg13398291	0.0741	-3.684	0.0556	-4.103	1.521
SFRP1	cg13154925	0.5821	0.483	0.5084	0.044	1.551
CDKN2A	cg12840719	0.0947	-3.304	0.0751	-3.670	1.443
CDKN2B	cg19233179	0.0686	-3.881	0.0497	-4.272	1.478
RASSF5	cg18328206	0.0430	-4.543	0.0544	-4.171	0.689
RASSF1	cg27149285	0.0348	-4.825	0.0278	-5.145	1.377
DAPK1	cg20401521	0.0325	-4.919	0.0395	-4.632	0.751
DAPK1	cg18387231	0.8412	2.421	0.8097	2.103	1.374
DAPK1	cg05365878	0.8687	2.747	0.8403	2.423	1.383
TP73	cg06996273	0.2369	-1.825	0.3086	-1.195	0.532
TP73	cg25885108	0.1205	-2.890	0.1410	-2.624	0.766
MGMT	cg20778669	0.7940	1.978	0.8383	2.400	0.656
MGMT	cg20537325	0.8923	3.079	0.9133	3.433	0.702
MGMT	cg18651291	0.8965	3.126	0.9149	3.462	0.714
MGMT	cg18485261	0.8291	2.284	0.8116	2.110	1.190
MGMT	cg11019008	0.7125	1.317	0.6845	1.120	1.218
MGMT	cg18502933	0.8097	2.098	0.7861	1.887	1.236
MGMT	cg13272119	0.7684	1.744	0.7403	1.519	1.252
MGMT	cg24810646	0.8783	2.866	0.8535	2.555	1.364
MGMT	cg24755725	0.8461	2.477	0.8130	2.145	1.394
MGMT	cg17083390	0.8747	2.834	0.8457	2.474	1.433
MGMT	cg04271445	0.7595	1.674	0.7088	1.305	1.445
MGMT	cg00904483	0.7984	2.020	0.7487	1.613	1.502
MGMT	cg18453665	0.7562	1.652	0.6965	1.224	1.534
GSTP1	cg23725454	0.1160	-3.001	0.1425	-2.612	0.677
GSTP1	cg26250609	0.0267	-5.240	0.0201	-5.710	1.601
MLH1	cg04841293	0.0404	-4.589	0.0332	-4.873	1.328
NKX6-2	cg03905847	0.1268	-2.881	0.1768	-2.289	0.553
NKX6-2	cg01384488	0.1078	-3.060	0.0842	-3.465	1.500
NKX6-2	cg08441806	0.1081	-3.107	0.0808	-3.550	1.557
TBX15	cg08857095	0.7334	1.465	0.7586	1.661	0.822
TBX15	cg27517681	0.2236	-1.856	0.1742	-2.262	1.501
DRD4	cg00556112	0.0539	-4.145	0.0451	-4.424	1.321
DRD4	cg03855291	0.4116	-0.536	0.3462	-0.929	1.481

**Table 5:** Number of CpG methylation sites with potential differences detected across HCC status by candidate gene and function

Function	Gene	Number of differentially methylated CpG sites^*^
**Tumor suppressor**	**PRDM2**	5
	**RUNX3**	5
	**RARB**	4
	**SFRP1**	3
	**CDKN2A (p16/p14)**	1
	**CDKN2B (p16/p14)**	1
	**RASSF5**	1
	**RASSF1**	1
**Cell cycle regulation**	**DAPK1**	3
	**TP73**	2
**DNA protection and repair**	**MGMT**	13
	**GSTP1**	2
	**MLH1**	1
**Tissue differentiation**	**NKX6-2**	3
	**TBX15**	2
**Other**	**DRD4**	2

* Not corrected for multiple comparisons.

## Discussion

Our study showed that saliva samples contained DNA of adequate volume and quality to detect methylation at CpG sites previously associated with HCC. We identified three CpG sites with suggestive increases in methylation in persons with HCC compared with control subjects, which would down-regulate expression of the encoded tissue differentiation, tumor suppression, and DNA repair proteins. Although this small study lacks the sample size to compare methylation patterns as biomarkers of disease or to adjust for potential confounders, it is nevertheless an important demonstration of the feasibility of using saliva in the next generation of DNA-based cancer screening (Tables 4 and 5).

In the case of colorectal cancer, the addition of screening tests that patients self-collect at home and deliver by mail (i.e., fecal immunohistochemistry testing or FIT, Cologuard) improved screening rates among persons not up to date with screening by colonoscopy [31]. The benefits of at-home test collection may be greater in rural communities with limited access to health facilities [32,33]. Given these demonstrated benefits of at-home screening tests and the ease of saliva collection for patients [18–20], this feasibility study provides preliminary evidence that salivary DNA methylation warrants further study.

Many biomarkers currently under study for prognostication after HCC diagnosis, development of targeted treatments, and screening for HCC in persons known to be at risk utilize epigenetic markers [5,6,34]. There is also potential for cell-free DNA (cfDNA) methylation markers to inform HCC risk stratification prior to cancer development. One study demonstrated the feasibility of such an application of cfDNA by analyzing repeated blood samples collected prior to HCC diagnosis in a Taiwanese cohort and identifying aberrant methylation in serum DNA between 1 and 9 years prior to HCC diagnosis [35]. In a study of genome-wide DNA methylation and copy number variation in regenerative nodules within individual livers, another study found that nodules demonstrating aggressive features were enriched for epigenetic changes associated with liver cancer, further supporting the possibility of using DNA methylation as a marker of early carcinogenesis [36].

Genetic and cellular material from liver parenchyma and tumor enters circulating blood through rich networks of hepatic sinusoids, which filter toxins and nutrients from blood reaching the liver through the portal vein and subsequently deliver blood to the systemic circulation through the hepatic veins. Both DNA from tumor and from at-risk liver parenchyma, which may exhibit more diffuse aberrations in DNA methylation as described above, may contribute to circulating DNA in the bloodstream and enter the saliva through the close interactions between capillary beds and salivary glands [23–25].

Importantly, in this study we were not able to assess potential confounding of the association between DNA methylation in saliva and HCC status by tobacco and alcohol consumption. Both of these exposures are associated with salivary DNA methylation changes and have well established associations with multiple cancers [37,38].

As the population at-risk of HCC changes over time, HCC risk stratification will encompass a more diverse patient population and will need to become more personalized [39]. If targeted panels of methylation markers can be identified and produced at-scale for specific populations, saliva-based DNA methylation testing may be a practical way to leverage these scientific advances in clinical care.

## Testable hypotheses and direction for future research

Multiple testable hypotheses are generated from this preclinical exploratory work. The overarching aims of future research should be to identify potentially useful biomarkers for further study from salivary DNA and estimate their accuracy (true positive rate, false positive rate, and receiver operating characteristic curve) [40]. Thus, early future research on salivary DNA methylation as a potential tool for HCC screening should center on testing the following hypotheses: Hypothesis 1: Salivary cfDNA methylation patterns accurately differentiate between individuals with HCC and individuals without HCC; Hypothesis 2: The pattern of DNA methylation changes associated with HCC status is not uniform across etiology of underlying liver disease. If the above hypotheses are supported by early evidence, additional clinical factors impacting biomarker performance should be assessed retrospectively in longitudinal biorepositories.

## Data Availability

Data utilized in this study include identifiable patient information. De-identified data are available upon request from the senior author.

## Open Access

Open access for this article was enabled by the participation of Yale University in an all-inclusive *Read & Publish* agreement with Portland Press and the Biochemical Society under a transformative agreement with Individual.

## Abbreviations

AFP: alpha-fetoprotein
cfDNA: cell-free DNA
ctDNA: circulating tumor DNA
FIT: fecal immunohistochemistry testing
HCC: hepatocellular carcinoma
HCV: hepatitis C virus
MASLD: metabolic dysfunction-associated steatotic liver disease
